# Evolutionary Dynamics of Begomoviruses and Its Satellites Infecting Papaya in India

**DOI:** 10.3389/fmicb.2022.879413

**Published:** 2022-05-12

**Authors:** Aarshi Srivastava, Vineeta Pandey, Anurag Kumar Sahu, Dinesh Yadav, Abdullah M. Al-Sadi, Muhammad Shafiq Shahid, R. K. Gaur

**Affiliations:** ^1^Department of Biotechnology, D.D.U. Gorakhpur University, Gorakhpur, India; ^2^International Center for Genetic Engineering and Biotechnology, New Delhi, India; ^3^Department of Plant Sciences, College of Agricultural and Marine Sciences, Sultan Qaboos University, Al-Khod, Oman

**Keywords:** papaya leaf curl diseases, phylogenetic analysis, recombination, genetic variability, population structure

## Abstract

The genus *Begomovirus* represents a group of multipartite viruses that significantly damage many agricultural crops, including papaya, and influence overall production. Papaya leaf curl disease (PaLCD) caused by the complex begomovirus species has several important implications and substantial losses in papaya production in many developing countries, including India. The increase in the number of begomovirus species poses a continuous threat to the overall production of papaya. Here, we attempted to map the genomic variation, mutation, evolution rate, and recombination to know the disease complexity and successful adaptation of PaLCD in India. For this, we retrieved 44 DNA-A and 26 betasatellite sequences from GenBank reported from India. An uneven distribution of evolutionary divergence has been observed using the maximum-likelihood algorithm across the branch length. Although there were phylogenetic differences, we found high rates of nucleotide substitution mutation in both viral and sub-viral genome datasets. We demonstrated frequent recombination of begomovirus species, with a maximum in intra-species recombinants. Furthermore, our results showed a high degree of genetic variability, demographic selection, and mean substitution rate acting on the population, supporting the emergence of a diverse and purifying selection of viruses and associated betasatellites. Moreover, variation in the genetic composition of all begomovirus datasets revealed a predominance of nucleotide diversity principally driven by mutation, which might further accelerate the advent of new strains and species and their adaption to various hosts with unique pathogenicity. Therefore, the finding of genetic variation and selection emphases on factors that contribute to the universal spread and evolution of Begomovirus and this unanticipated diversity may also provide guidelines toward future evolutionary trend analyses and the development of wide-ranging disease control strategies for begomoviruses associated with PaLCD.

## Introduction

Begomoviruses (family: *Geminiviridae*) are the largest group of plant viruses that pose a significant yield loss to many economically important crops in tropical and sub-tropical regions of the world (Varma and Malathi, 2003). Begomoviruses are transmitted by the whitefly *Bemisia tabaci*, a cryptic species complex which has been adapted and co-evolved with the *begomovirus* genome (Brown et al., 2012; Marwal et al., 2021). Due to its semi-persistent mode of transmission and behavioural manipulation, *B. tabaci* is considered the second most important vector of plant viruses that effectively transmits a large number of virus species belonging to the genus begomovirus (Moreno-Delafuente et al., 2013).

**Figure 1:**
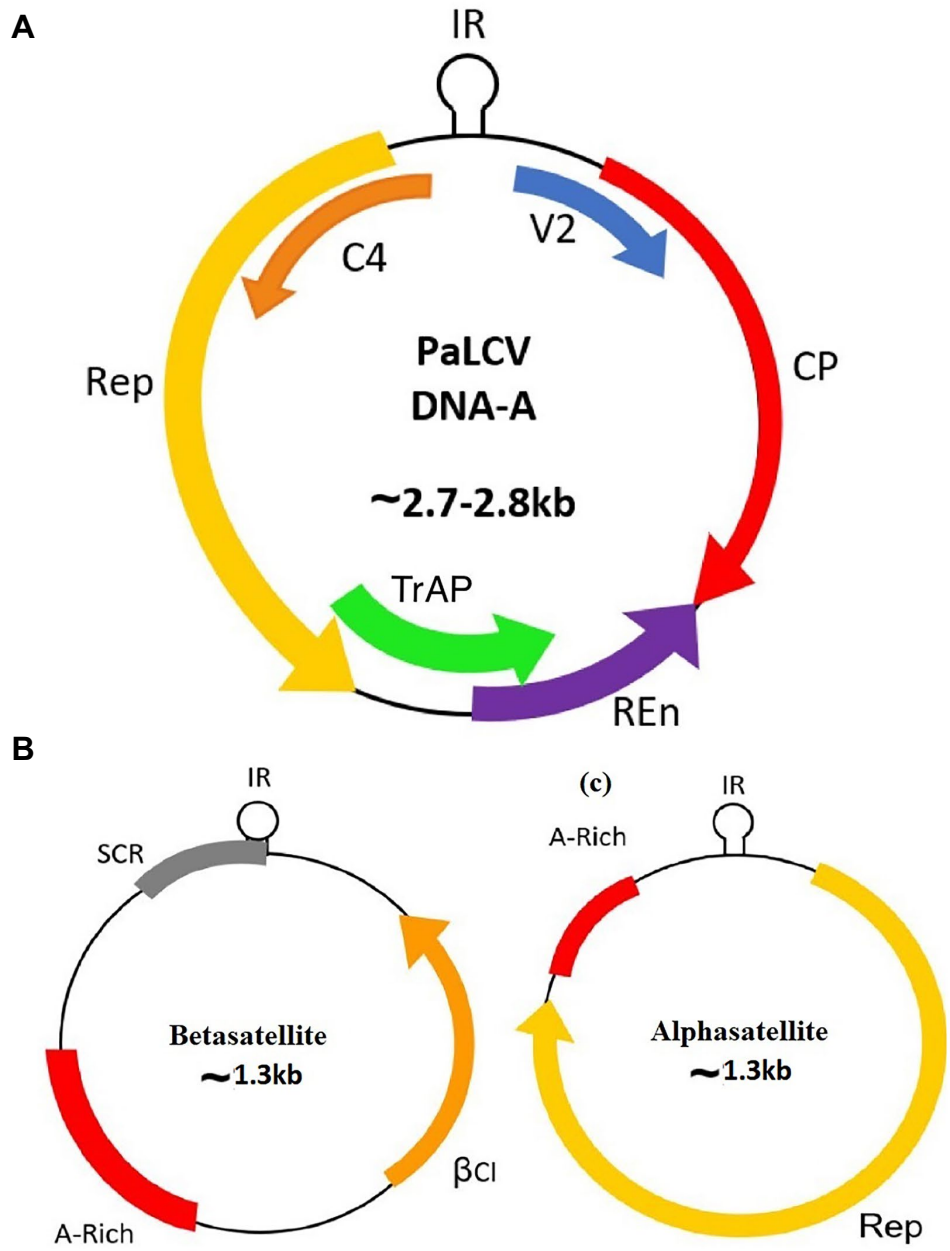
Genome organisation of the Papaya Leaf Curl Virus with associated satellites. **(A)** DNA-A; **(B)** Betasatellite; and **(C)** Alphasatellite.

**Figure 2:**
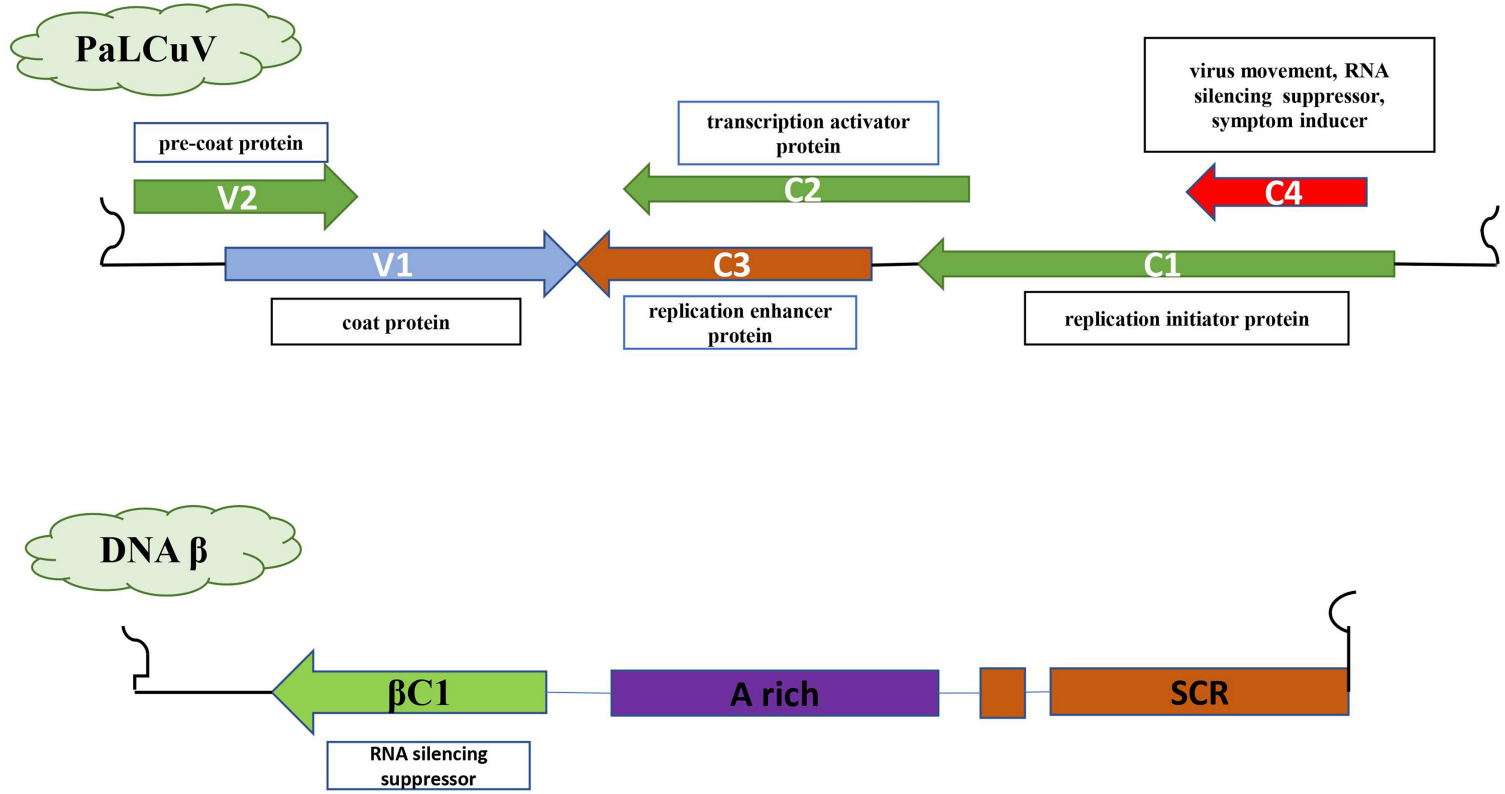
The genomic component of the monopartite Papaya Leaf Curl Virus genome with betasatellite indicating the silencing suppressor genes; six ORF (AV2, AC2, AC4, AV1, AC3, and AC1); βC1.

Begomoviruses are approximately 2.7–2.8 Kb in size and composed of circular single-stranded DNA (ssDNA) encapsulated in a quasi-isometric non-enveloped twinned particle (Stanley et al., 2005). Begomoviruses are either bipartite [native to New World (NW)] segments of DNA A and DNA B or monopartite [native to Old World (OW)] segment of DNA A. The OW monopartite begomoviruses are also associated with ssDNA helper molecules designated as betasatellite or alphasatellite and a newly reported deltasatellite (Zhou, 2013; Lozano et al., 2016). Papaya leaf curl virus (PaLCuV) is a monopartite begomovirus ~2.7–2.8 Kb in size (Figure 1) that causes PaLCD. PaLCuV genome consists of six open reading frames (ORFs) systematised in two transcriptional directions and are divided by an intergenic region (IR) (Nehra et al., 2019). They have been named according to their functions, and encode proteins (AV2, AV1, AC3, AC2, AC1, and AC4) that assist in viral particles movement (intra- and inter-cellular) within the host. Besides, this IR contains a highly conserved unique nonanucleotide “TAATATTAC” with an origin of replication (Figure 2). Moreover, OW monopartite viruses are mostly associated with betasatellite, which encodes an important βC1 protein (Figure 2) at the complementary sense strand and plays an important role in transcriptional and post-transcriptional gene silencing, disease epidemics, and symptom induction (Varun and Saxena, 2018), and can also affect the Jasmonic acid repressor gene (Zhou, 2013). Thus, begomovirus together with betasatellite causes systemic infection and develops typical symptoms in plants (Nawaz-ul-Rehman and Fauquet, 2009; Hanley-Bowdoin et al., 2013). Leaf curl symptoms caused by the *papaya leaf curl virus* are found associated with infected papaya plants from different regions, which initiate serious production losses and can act as a potential inducer for viral transmission *via* the vector whitefly (Guo et al., 2015).

**Figure 3:**
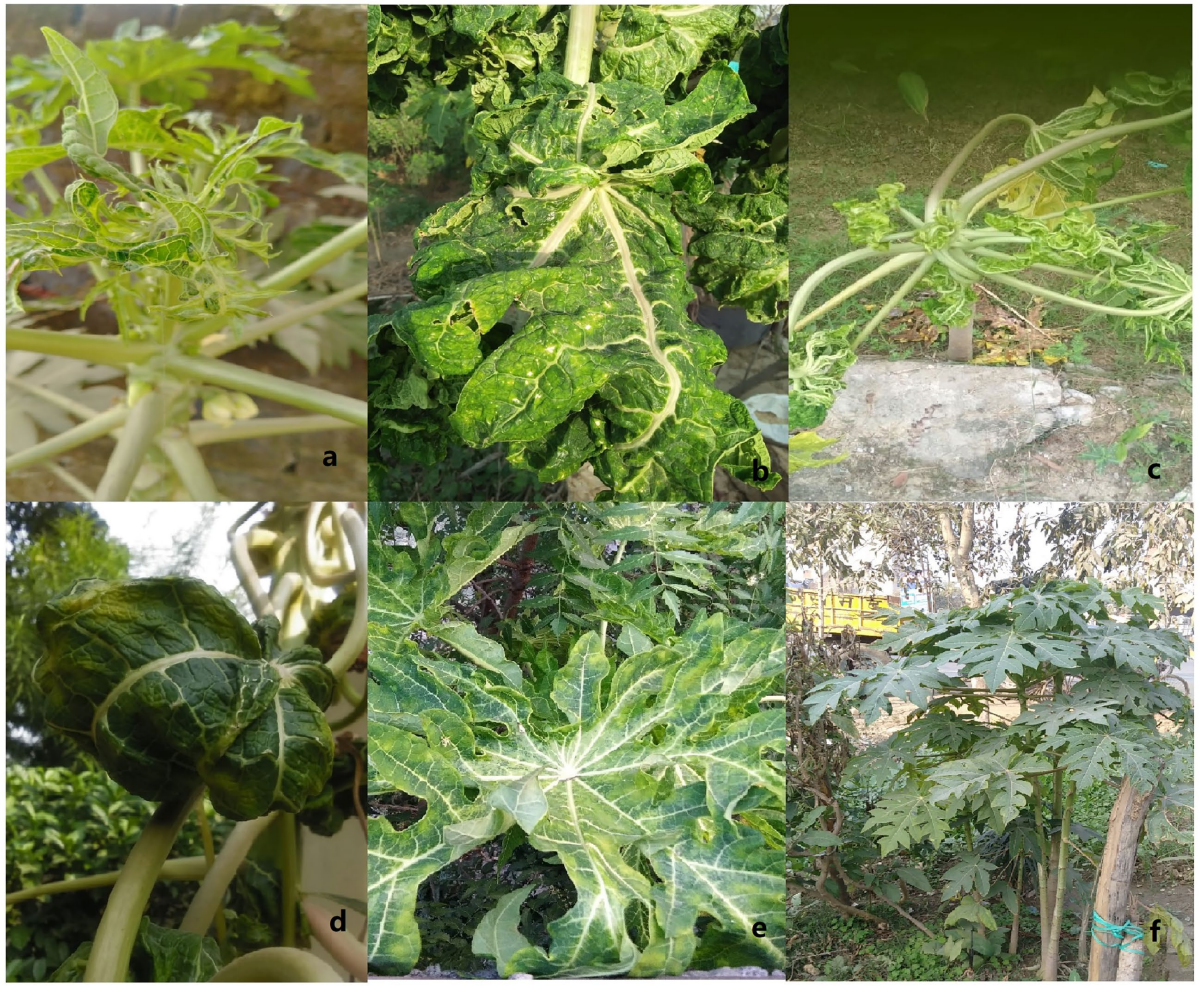
Observed symptoms in *Carica papaya* infected with PaLCD begomoviruses. Severe upward and downward curling, thickening, crinkling, and yellowing of leaves **(A–E)** along with healthy leaves **(F)**.

Papaya (*Carica papaya*), a major tropical, sweet, large, and herbaceous food crop, belongs to the order Brassicales (family: *Caricaceae*) and is cultivated throughout India (Yadav et al., 2017). However, its local and commercial cultivation makes it difficult to achieve its full potential (FAOSTAT, 2019) due to the large number of insects pest, bacteria, fungi, and viruses. Among viral diseases, papaya leaf curl disease (PaLCD) caused by a complex of begomovirus species is a major damaging factor, which affects the overall production of papaya (Nascimento et al., 2010). PaLCD caused by a geminivirus was first reported by Thomas and Krishnaswamy in 1939 and, subsequently, the causative virus was further confirmed by Saxena et al. (1998). Since then, the number of begomovirus species infecting papaya crops has been increasing. Begomovirus infected papaya plants show typical symptoms of begomovirus infection, including yellowing, curling, leaf distortion, and stunting (Figure 3). India, a leading papaya-producing country in the world, accounts for a severe infection impact of PaLCuV, including other begomovirus species, which impedes commercial papaya production of papaya plants, and moreover, it could also reduce the growth of the pharmaceutical industry. Therefore, infection of begomovirus associated with PaLCD causes greater loss to the papaya crop, which not only had anatomical and physiological losses, but their pharmacological potential was also significantly diminished (Soni et al., 2022). In India, PaLCD is associated with a complex of 15 begomovirus species, such as *Papaya leaf curl virus* (PaLCuV), *Papaya leaf crumple virus* (PaLCrV), *Chilli leaf curl virus* (ChiLCV), *Chilli leaf curl India virus* (ChiLCINV), *Duranta leaf curl virus* (DLCV), *Papaya yellow leaf curl virus* (PaYLCV), *Papaya severe leaf curl virus* (PaSLCV), *Tomato leaf curl New Delhi virus* (ToLCNDV), *Tomato leaf curl virus* (ToLCV), *Cotton leaf curl Multan virus* (CLCuMuV), *Tomato leaf curl Gujarat virus* (ToLCGV), *Croton yellow vein mosaic virus* (CYVMV), *Pedilanthus leaf curl virus* (PeLCV), *Ageratum enation virus* (AEV), and *Cotton leaf curl Burewala virus* (CLCuBuV).

In spite of being an economically important tropical fruit, little attention has been paid to assessing the genetic diversity at the molecular level of the papaya infecting begomoviruses (Fougat et al., 2015). The current information about the geographical distribution in India, genetic diversity, and evolutionary dynamics of begomovirus and associated satellites for papaya infecting plants remains insufficient or little information is available. Moreover, there is no data available regarding comparative analysis of genetic variability and evolutionary aspects to understand the virus and satellite populations arising from different geographical locations in India. In the present study, we focused on the current diversity of PaLCD-causing begomoviruses in India, genomic variability, population dynamics, and evolutionary patterns to get further insights into the complexity of PaLCD begomoviruses and their accompanying satellites.

## Materials and Methods

### Sequence Retrieval and Sequence Alignment

**Figure 4:**
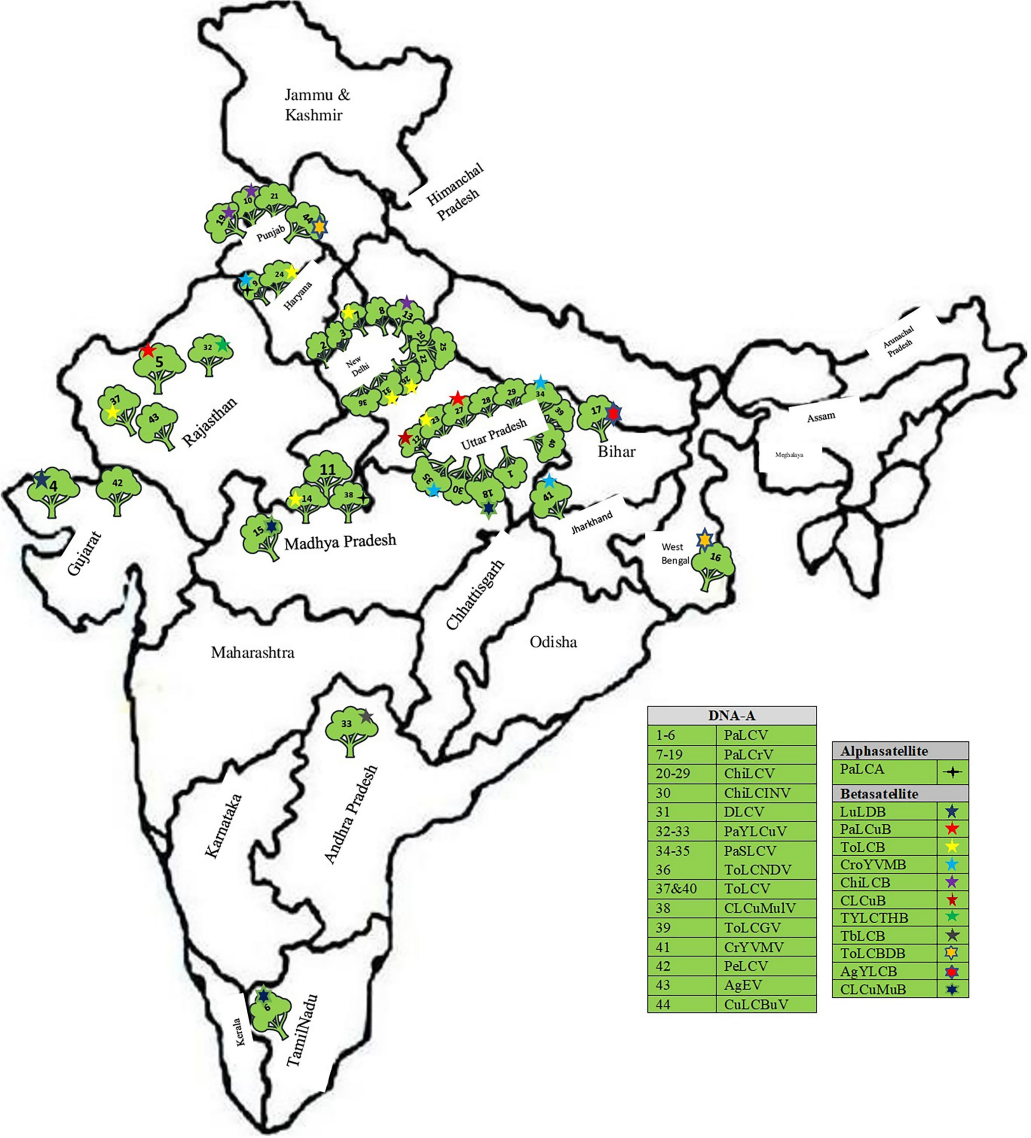
Distribution of distinct begomoviruses isolates and their satellites in India associated with leaf curl disease of *Carica papaya* (Follow Table 1).

A total of 70 complete genomic sequences of begomoviruses and their associated satellites infecting papaya plants were retrieved from GenBank,^1^ including 44 sequences of DNA-A and 26 sequences of betasatellite (Figure 4; Table 1). The sequences of all begomoviruses and sub-viral satellites associated with PaLCD reported till June 30, 2021, were included in the study to explore its diversity and distribution in India. A total of eight datasets were formed (DNA-A, its six ORFs, and betasatellite), and each specific dataset was aligned through a multiple sequence alignment algorithm with Clustal W (Thompson et al., 1994), using MEGA X software (Kumar et al., 2018). Only two alphasatellites sequences associated with the papaya host were found at the NCBI gene data bank, which is not sufficient to show significant analysis and, therefore, we have not included them in sequence analysis in this study. All sequences were organised to begin at the nick site of the conserved nonanucleotide motif at the origin of replication (5′-TAATATT//AC-3′).

**Table 1:** Features of *begomoviruses* and associated satellites causing leaf curl disease in papaya plants are identified in India.

Accession No.	*Begomoviruses*	Genome nature	Isolate	Year	Betasatellite(s)		Alphasatellite(s)	
Y15934	*Papaya leaf curl virus*	Monopartite	0	1997	0	0	0	0
KF307208	*Papaya leaf curl virus*	Monopartite	Pap:ND:13	2013	0	0	0	0
KY800906	*Papaya leaf curl virus*	Monopartite	IN/ND/Pap/16	2017	0	0	0	0
MH807205	*Papaya leaf curl virus*	Monopartite	PSB-34	2018	MH825685	LuLDB	0	0
MN529626	*Papaya leaf curl virus*	Monopartite	MM1	2019	MN529627	PaLCuB	0	0
KX302713	*Papaya leaf curl virus*	Monopartite	Wellington	2010	KX302720	CLCuMuB	0	0
HM140369	*Papaya leaf crumple virus*	Monopartite	Naj1[IN:ND:Pap:08]	2010	HM143909	ToLCB	0	0
HM140368	*Papaya leaf crumple virus*	Monopartite	Nir [IN:ND:Pap:07]	2010	0	0	0	0
HM140367	*Papaya leaf crumple virus*	Monopartite	Pani8[IN:Pani:Pap:08]	2010	HM143908	CroYVMB	0	0
KR052159	*Papaya leaf crumple virus*	Monopartite	Mohali	2015	KR052158	ChiLCB	KR052157	PaLCA
MH674437	*Papaya leaf crumple virus*	Monopartite	PSB-32	2018	0	0	0	0
MH807200	*Papaya leaf crumple virus*	Monopartite	PSB-47	2018	MH825687	CLCuB	0	0
MH807201	*Papaya leaf crumple virus*	Monopartite	PSB-60	2018	MH825689	ChiLCB	0	0
MH807203	*Papaya leaf crumple virus*	Monopartite	PSB-66	2018	MH825691	ToLCB	0	0
KX302712	*Papaya leaf crumple virus*	Monopartite	Bhopal	2011	KX302719	CLCuMuB	0	0
KX302711	*Papaya leaf crumple virus*	Monopartite	Kolkata	2012	KX302715	ToLCBDB	0	0
KX302710	*Papaya leaf crumple virus*	Monopartite	Hajipur	2011	KX302714	AgYLCB	0	0
KX302709	*Papaya leaf crumple virus*	Monopartite	Lucknow	2012	KX302718	CLCuMuB	0	0
KX302708	*Papaya leaf crumple virus*	Monopartite	Mohali	2012	KX302717	ChiLCB	0	0
DQ989326	*Chilli leaf curl virus*	Monopartite	AD	2006	0	0	0	0
GU136803	*Chilli leaf curl virus*	Monopartite	IN:Amrit:Pap:09	2009	0	0	0	0
HM140371	*Chilli leaf curl virus*	Monopartite	Noida	2010	0	0	0	0
HM140370	*Chilli leaf curl virus*	Monopartite	Najafgarh-2	2010	HM143911	ToLCB	0	0
HM140366	*Chilli leaf curl virus*	Monopartite	Panipat-1	2010	HM143901	ToLCB	0	0
HM140365	*Chilli leaf curl virus*	Monopartite	HD	2010	0	0	0	0
HM140364	*Chilli leaf curl virus*	Monopartite	DU	2010	HM143910	ToLCB	0	0
MH765693	*Chilli leaf curl virus*	Monopartite	PSB-21	2018	MH825684	PaLCuB	0	0
MH765697	*Chilli leaf curl virus*	Monopartite	PSB-42	2018	0	0	0	0
MH765698	*Chilli leaf curl virus*	Monopartite	PSB-45	2018	0	0	00	0
MF574143	*Chilli leaf curl India virus*	Monopartite	Meerut	2017	0	0	0	0
MH807202	*Duranta leaf curl virus*	Monopartite	PSB-63	2018	MH825690	ToLCB	0	0
KX353622	*Papaya yellow leaf curl virus*	Monopartite	DP2	2017	KX353620	TYLCTHB	0	0
MH807204	*Papaya yellow leaf curl virus*	Monopartite	PSB-51	2018	MH825688	TbLCB	0	0
MH988458	*Papaya severe leaf curl virus*	Monopartite	PSB-14	2018	MH825683	CroYVMB	0	0
MH988457	*Papaya severe leaf curl virus*	Monopartite	PSB-8	2014	HM143908	CroYVMB	0	0
DQ989325	*Tomato leaf curl New Delhi virus*	Monopartite	PD	2006	0	0	0	0
KP725055	*Tomato leaf curl virus*	Monopartite	C1	2014	KP725056	ToLCB	0	0
JN558352	*Cotton leaf curl Multan virus*	Monopartite	CLCuV	2011	0	0	JQ322970	PaLCA
MG757245	*Tomato leaf curl Gujarat virus*	Monopartite	LUCKNOW	2018	0	0	0	0
MH105055	*Tomato leaf curl virus*	Monopartite	Sultanpur	2018	0	0	0	0
MH765696	*Croton yellow vein mosaic virus*	Monopartite	PSB-38	2018	MH825686	CroYVMB	0	0
MH765695	*Pedilanthus leaf curl virus*	Monopartite	PSB-37	2018	0	0	0	0
KP725057	*Ageratum enation virus*	Monopartite	CN2	2014	0	0	0	0
KX302707	*Cotton leaf curl Burewala virus*	Monopartite	Guntur	2011	KX302716	ToLCBDB	0	0

### Phylogenetic Analysis and Detection of Substitution Mutation Bias

A phylogenetic analysis was performed using the MEGA X program with bootstrap analysis utilising 1,000 replicates. The evolutionary history for sequence datasets DNA-A, its six ORFs and associated betasatellites was calculated using the maximum likelihood (ML) tree based on CLUSTAL W pairwise alignment and the best fit nucleotide substitution model, i.e., (GTR + G) for DNA-A, its ORFs (AV1: TN93 + G + I); (AV2: HKY + G); (AC1 and AC3: GTR + G); (AC2: TN93 + G); (AC4: HKY + G), and (TN93 + G + I) for betasatellites, based on the corrected Bayesian Information Criterion (BIC) score of the MEGA X program (Kumar et al., 2018). Moreover, the transversion and transition DNA substitution rates as well as the transition/transversion bias (R) were also calculated for the virus, its ORFs and betasatellites using MEGA X (Kumar et al., 2018).

### Detection of Recombination

The recombination analysis was done by using seven methods, i.e., RDP (Martin and Rybicki, 2000), BOOTSCAN (Martin and Williamson, 2005), CHIMAERA (Posada and Crandall, 2001), GENECONV (Padidam et al., 1999), MAXCHI (Maynard, 1992), 3SEQ (Boni et al., 2007), and SISCAN (Gibbs et al., 2000) implemented in the recombination detection program (RDP) v.4.1 software (Martin et al., 2015). For analysis, toward 0.05, the highest acceptable Bonferroni corrected value of *p* and default detection thresholds, datasets were subjected. To reduce false-positive results, out of seven, at least three algorithms were considered appropriate to detect recombination events.

### Coalescent Analysis

The rates of nucleotide substitution per site and the rates of mutation at three different codon positions (C1, C2, and C3) in the sequence datasets of begomoviruses, their ORFs and betasatellite were determined by using the Bayesian Markov Chain Monte Carlo (MCMC) parameter of Bayesian Evolutionary Analysis Sampling Tree (BEAST; v.1.10; Suchard et al., 2018). Coalescent constant demographic models and best-fit molecular clock were detected using BEAST, whose sample size was effectively achieved through the use of the Tracer program (v.1.5; Rambaut et al., 2018). The MCMC chain used a 10% burn-in value with 10^7^ run lengths to provide a 95% highest probability density (HPD) interval for determining statistical uncertainty. For assessing temporal structure, which is important for the estimation of substitution rates, we repeated the BEAST analysis by reshuffling the sampling times for each dataset, which were randomised on the tips (Ramsden et al., 2008), and then the HPDs of these randomised sequences were compared with those of the real data.

### The Population Structure Assay

To determine the genetic diversity of a virus population, several parameters of the DnaSP (v. 6.12) software were used (Rozas et al., 2017). The aligned sequence datasets were examined for the total number of segregating sites (s), the average number of nucleotide differences between sequences (k), the total number of mutations (η), the nucleotide diversity (π), and Watterson’s estimate of the population mutation rate based on the total number of segregating sites (θ − w) and the total number of mutations (θ – η) were also calculated along with a number of haplotypes (h), and haplotype diversity (Hd; Lima et al., 2017).

The Neutrality Test is executed to calculate the hypothesis of selection pressure occurring in the population using DnaSP (v.6.12) software (Rozas et al., 2017; Universitat de Barcelona; origin 1994). Therefore, sequence datasets separated by different geographical locations are tested by employing tests such as Tajima’s *D*, which signifies the difference between the two measures of genetic diversity, i.e., the number of segregating sites and the mean number of pairwise differences, Fu and Li’s D* for identifying the difference between the total number of mutations and the number of singletons, and Fu and Li’s F* for identifying the difference between the average number of nucleotide differences between paired sequences and the number of singletons, i.e., detecting the neutrality of mutations in DNA populations (Rozas et al., 2017).

## Results

### Geographical Distribution, Phylogenetic Analysis, and Detection of Substitution Mutation Bias

**Figure 5:**
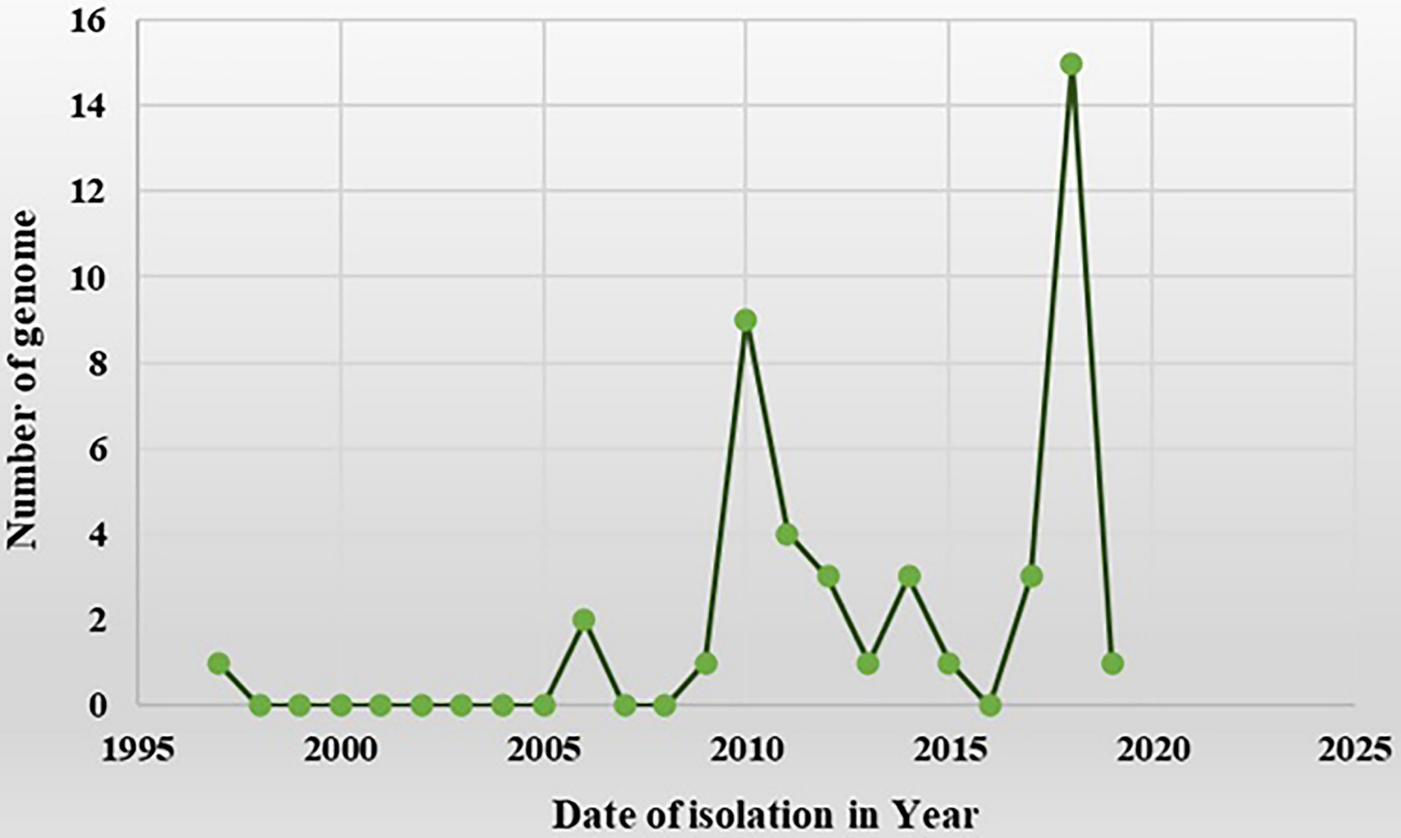
A time-curve graph depicting the distribution of 44 begomovirus genomes in India by date of isolation. The numbers of complete genomes of begomovirus were plotted against the time of their collection from the year 1997 to the year 2021 (data from NCBI).

The complete genomic sequence of PaLCD-associated begomoviruses and their satellites was considered in the current study, which included a viral genome of 2.7–2.8 Kb in size and an associated satellite of 1.3 Kb in size (Figure 2). The total of 44 begomovirus isolates of Indian origin showed the wide distribution of the viruses, which predominantly constitute six sequences of *Papaya* leaf curl virus (PaLCV), 13 sequences of *Papaya* leaf crumple *virus* (PaLCrV), and 10 sequences of *Chilli* leaf curl *virus* (ChiLCV), infecting papaya plants in India (Table 1). Moreover, a total of 26 betasatellite isolates, which predominantly constitute seven sequences of Tomato leaf curl betasatellite (ToLCB), four sequences of Croton yellow vein mosaic betasatellite (CroYVMB), and three sequences of each for Cotton leaf curl Multan betasatellite (CLCuMuB) and Chilli leaf curl betasatellite (ChiLCB) respectively, showed the mixture of their species infecting papaya plants as per Table 1. Only two papaya plant-associated alphasatellite isolates, i.e., papaya leaf curl alphasatellite (KR052157 and JQ322970) were recovered and were found insufficient to give significant results for the present study. The time curve distribution of 44 begomovirus genomes by date of isolation in India shows a large variation from the year 1997 to the year 2021 (Figure 5).

**Figure 6:**
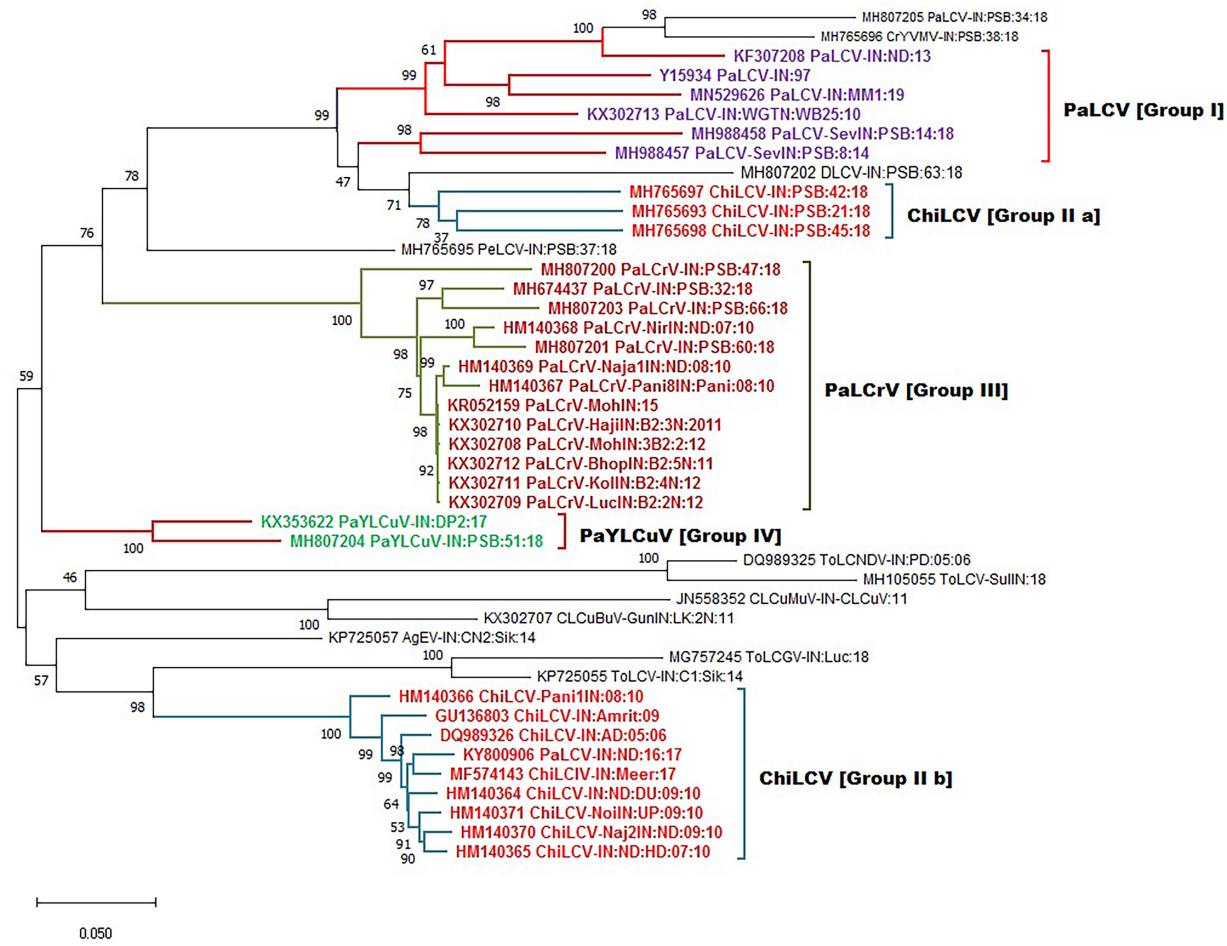
Maximum-likelihood phylogeny-based partitioning tree associated with PaLCD begomoviruses in India aligned using CLUSTAL W with a 1,000 bootstrap value within the MEGA v.10 program; 44 begomovirus isolates (DNA-A sequences) form four distinct major groups.

**Figure 7:**
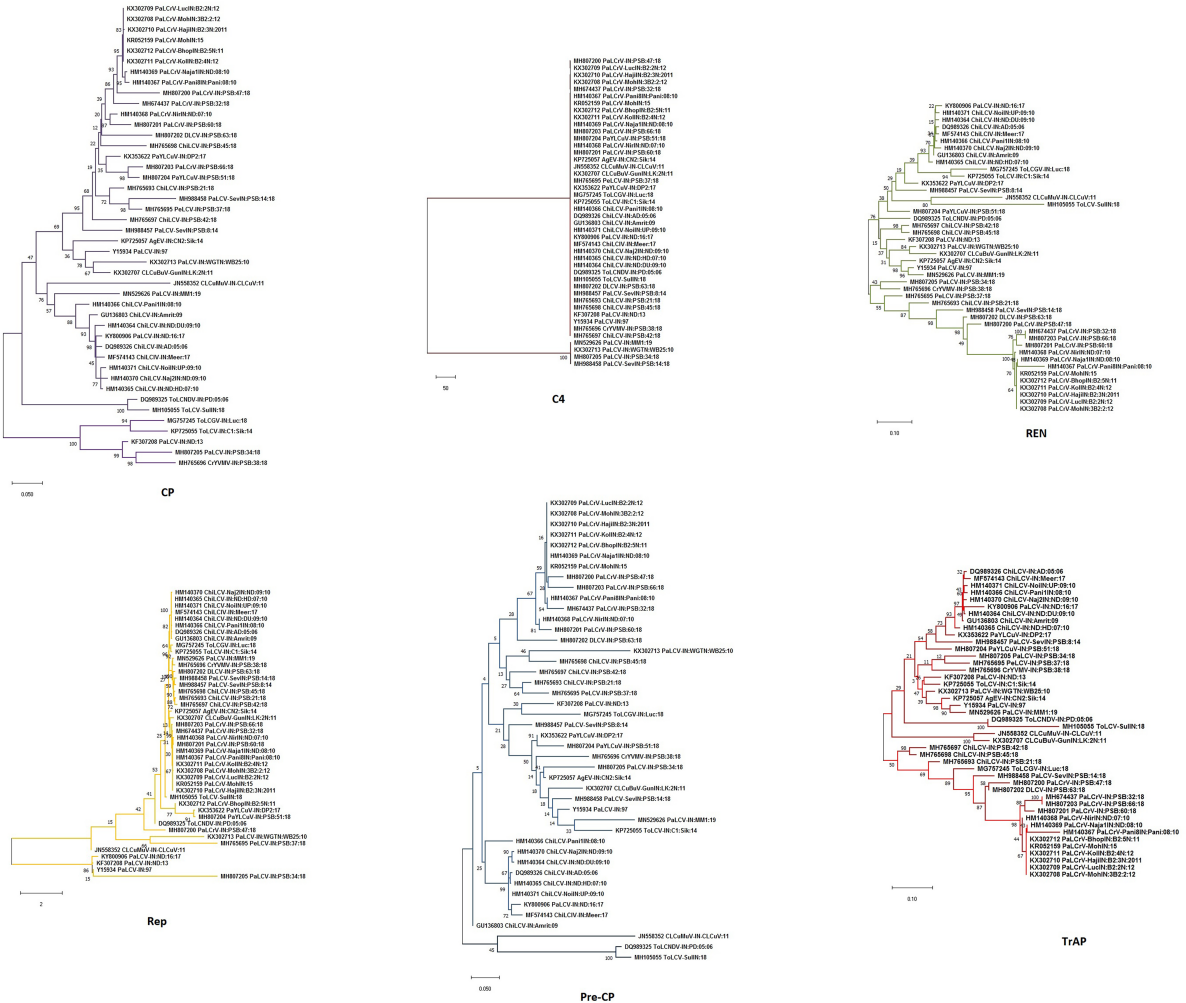
Maximum-likelihood phylogenetic dendrogram of all the six genes/ORFs of begomoviruses associated with PaLCD in India aligned using CLUSTAL W with a 1,000 bootstrap value within the MEGA v.10 program; six ORFs (CP; C4; REn; Rep; Pre-CP; and TrAp) of 44 begomovirus isolates.

After collecting the various begomovirus sequences associated with the papaya host from the NCBI GenBank, we used phylogenetic analysis to discover the interconnection between different begomovirus species evolving from different regions and clustering into a distinct group. The evolutionary history of DNA A sequence datasets was observed with four major distant lineages with 1,000 bootstrap support and were grouped as (PaLCuV I), (PaLCrV II), (PaYLCuV III), and (ChiLCV IV a & b; Figure 6). The lineages comprise the isolates collected from different geographic locations in India (Table 1). The branch length among a population suggests the level of differentiation within them. Group I showed clustering among isolates of PaLCuV from New Delhi, Gujrat, and the Rajasthan region. Group II of PaLCrV forms a cluster between the isolates of New Delhi, Haryana, Punjab, and some regions of Madhya Pradesh and Uttar Pradesh. Further, PaYLCuV forms group III and get clustered between the isolates of New Delhi and Rajasthan. The two groups of ChiLCV were observed in which one group of ChiLCV (IVa) shows clustering with the major group I, which suggests the intra-species similarity with PaLCuV, whereas the second ChiLCV group (IVb) forms a separate clade expressing their inter-species relations. Additionally, some small groups are also observed, clustering with their closely related species. Similarly, the phylogenetic analysis was also performed for six different ORFs of DNA-A and observed the sequence homology for selected genome regions (Figure 7).

**Figure 8:**
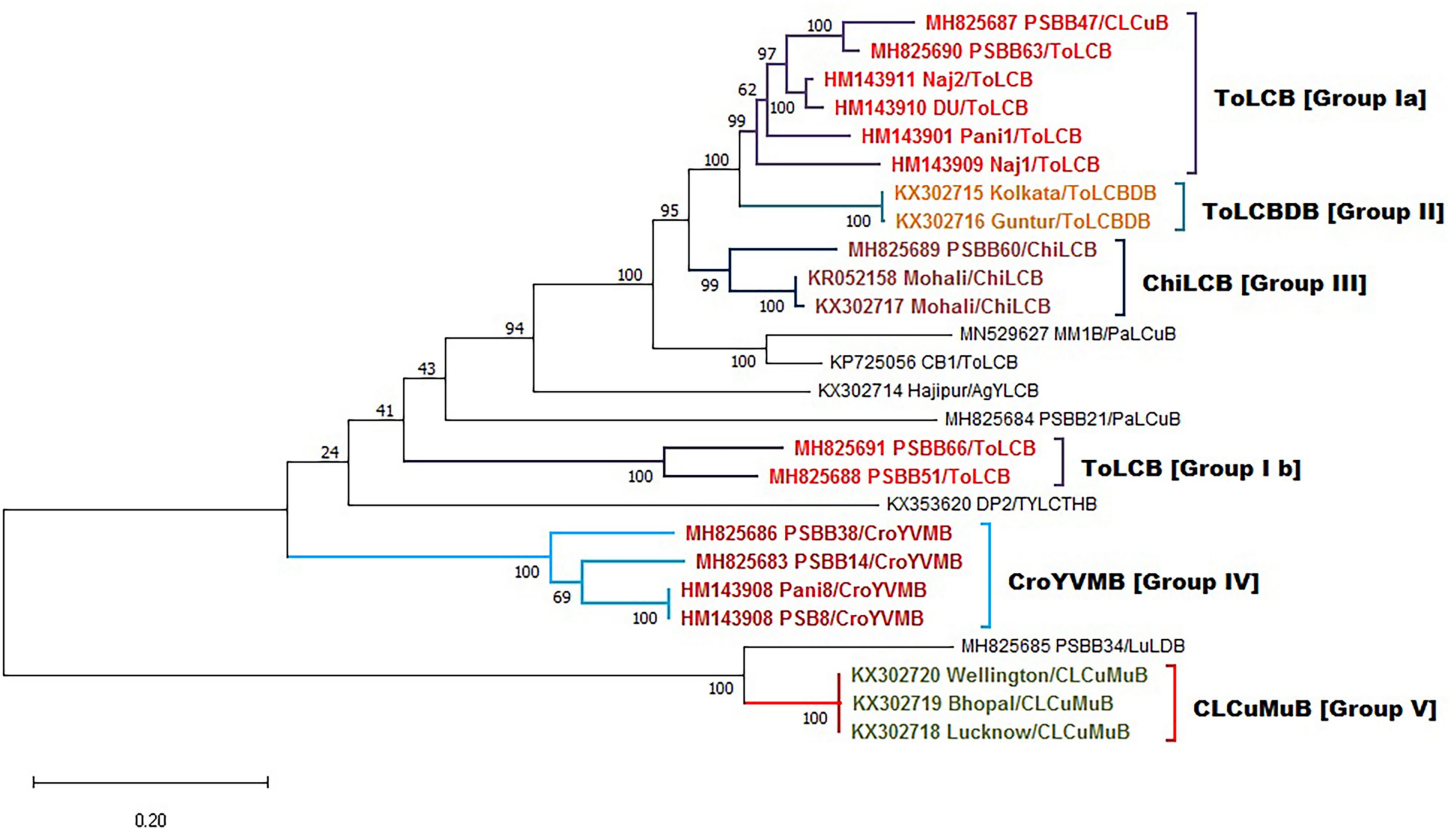
Maximum-likelihood phylogeny-based partitioning tree of begomoviruses linked betasatellites associated with PaLCD in India aligned using CLUSTAL W with 1,000 bootstrap value in the MEGA v.10 program; 26 betasatellites isolates for five distinct groups.

In addition, well-defined clusters are observed in the case of betasatellite, depicting five distinct groups as (ToLCB I a & b), (ToLCBDB II), (ChiLCB III), (CroYVMB IV), and (CLCuMuB V; Figure 8). Further, different rates of transition, transversion DNA substitution mutation, and transition/transversion bias (R) were estimated for the *begomovirus* isolates causing PaLCD (Table 2). However, in our study, we found that the rate of transition and transversion substitution mutation in genomic regions ranged from 18.99–27.34 to 1–1.44, respectively, while the transition/transversion bias (R) was 1.00. Further, independent analysis of six ORF datasets showed variable values for different substitution mutation rates. We found the transition/transversion rate for all six ORF regions is AV2, AC2, and AC4 regions are most susceptible to transition and less susceptible to transversion, while AC3 and AC1 are more prone to transversion than transition. Moreover, we found transition/transversion bias (R) ratios were close to 1 (Table 2), except in AC2 and AV1 regions where they were equal to 1. Therefore, a higher transition/transversion ratio, especially in AV1 and AC2 regions (1.0), suggests that either negative selection is removing transversions and transitions are favoured, whereas a lower transition/transversion ratio, especially in AV2, AC3, AC1, and AC4 regions (0.9), suggests that either negative selection is removing transitions and transversions are favoured. Similarly, for betasatellite, the transition and transversion substitution mutation range were obtained as 0.23–55.55 and 0.42–0.72, respectively, while the transition/transversion bias (R) was found at 0.81. However, we conclude that selection is a contributor towards transition/transversion bias (R) in begomoviruses, which cannot be partially explained by either transition or transversion value. Therefore, the estimation suggests that the role of base substitutions causes transition mutation at a higher rate in a population than single nucleotide polymorphism.

**Table 2:** Estimation of substitution rate among DNA-A, open reading frames (ORFs), and betasatellite associated with Papaya Leaf Curl Disease (PaLCD) in India using the MEGA X program.

Virus components	Transition substitution rate	Transversion substitutions rate	Transition/Transversion bias (R)
DNA-A	18.99–27.34	1–1.44	1.00
Betasatellite	0.23–55.55	0.42–0.72	0.81
AV2(Pre-CP)	10.19–15.19	5.73–6.54	0.95
AV1(CP)	8.36–31.42	2.77–3.78	1.17
AC3(Ren)	7.87–20.35	4.55–8.07	0.85
AC2(TrAP)	8.79–17.81	5.24–7.14	1.14
AC1(Rep)	9.3–13.4	5.85–8.43	0.96
AC4	10.07–27.95	3.33–4.66	0.90

### Detection of Recombination

Further, the tree-like phylogenetic divergence obtained for sequence datasets directed us to detect the occurrence of non-tree-like evolution within a population to explain the potential for recombination events in aligned sequences. Different parameters were used to determine the shared overlapping intra- and inter-specific recombination events distributed throughout the genome with different parental combinations. Forty-eight putative recombination events were observed for DNA-A datasets, in which 30 isolates were found to be recombinant. Out of 30 isolates, 20 had a single recombination event, whereas 10 showed multiple events of recombination (Table 3). The AC1 genome region DNA-A datasets are highly prone to recombination, exhibiting 19 putative breakpoints (1,527–2,612 nucleotide positions; Table 4), with 15 recombinants having major and minor parents. However, both intra and inter-specific recombination among different begomovirus isolates was predominately observed in the AC1, AV1, and AC2 rich genome regions, while the AV2, AC3, and AC4 genome regions were less susceptible to recombination. Additionally, 15 putative recombination breakpoints were identified for betasatellites, among which 12 isolates were found to be recombinant, of which eight isolates had a single recombination event and four isolates were found to have more than one event of recombination (Table 3). Moreover, recombination events distributed predominantly in the βC1 genome region of betasatellites support the prevalence of recombination that is involved in virus movement by suppressing host antiviral silencing genes (Kumar et al., 2015). Further, relevant recombination events were obtained by selecting at least three or more methods that minimise incompetent outcomes. Thus, significant amounts of genetic variation were supported by the maximum putative recombinational events among sequence datasets.

**Table 3:** Putative recombination events detected using the RDP4.1 program among *begomoviruses* and betasatellites associated with PaLCD, based on provided datasets from India; 44 DNA-A sequence and 26 associated betasatellite.

Event no.	Breakpoints		Recombinant	Parents		Methods	*p* valve
DNA-A	Begin	End		Major	Minor		
1	2,330	2,680	MH765693_ChiLCV	MH807200_PaLCr	Unknown (KP725057_AgEV)	**R**,G,M,C,S,3S	8.36E-49
2	458	1,186	MH807205_PaLCV	KX302713_PaLCV	Unknown (KX302707_CLCuB)	R,G,B,M,C,S,**3S**	1.91E-44
3	431	1,256	KX302707_CLCuBV	Unknown(MH765696_CrYVM)	KX302713_PaLCV	R,G,M,C,S,**3S**	8.12E-44
4	988	1,373	DQ989325_ToLCNDV	MH105055_ToLCV	KX302713_PaLCV	**R**,G,M,C,S,3S	2.84E-35
5	2,335	2,679	MH988458_PaLCV	KP725057_AgEV	Unknown (MH807200_PaLCr)	R,**G**,M,C,S,3S	7.60E-34
6	500	1,050	MH765696_CrYVMV	MH765693_ChiLC	Unknown (KX353622_PaYLC)	**R**,G,M,C,S,3S	4.27E-26
7	2,312	2,656	MH807200_PaLCrV	MH807202_DLCV	KP725057_AgEV	**R**,G,M,C,S,3S	3.01E-25
8	136	446	KX302713_PaLCV	KP725055_ToLCV	Unknown (MH988458_PaLCV)	**R**,G,B,M,C,S,3S	4.46E-24
9	1,292	1,365	HM140367_PaLCrV	HM140369_PaLCr	KF307208_PaLCV	**R**,G,M,C,3S	1.38E-21
10	334	1,274	MG757245_ToLCGV	KX353622_PaYLC	Unknown (MH807202_DLCV)	**R**,G,M,C,S,3S	3.02E-22
11	2,032	2,326	MH807204_PaYLCV	Unknown (MH765693_ChiLC)	HM140368_PaLCr	R,**G**,M,C,S,3S	6.75E-20
12	441	959	KX353622_PaYLCV	MN529626_PaLCV	HM140368_PaLCr	**R**,G,B,M,C,S,3S	3.06E-19
13	159	430	KX302707_CLCuBV	JN558352_CLCuM	MH988458_PaLCV	**R**,G,C,3S	3.60E-19
14	2,663	121	MH105055_ToLCV	Unknown (MH807202_DLCV)	MH807200_PaLCr	**R**,G,M,C,S,3S	1.03E-16
15	2,680	256	KF307208_PaLCV	MH765696_CrYVM	MH765693_ChiLC	**R**,G,M,S,3S	1.52E-15
16	2,110	2,325	MG757245_ToLCGV	MN529626_PaLCV	Unknown (MH765693_ChiLC)	**R**,G,M,C,S,3S	1.71E-14
17	2,109	2,328	HM140364_ChiLCV	MN529626_PaLCV	Unknown (MH765693_ChiLC)	R,G,M,C,**S**	9.38E-16
18	2,657	744	MH807200_PaLCrV	MH807202_DLCV	HM140369_PaLCr	R,G,M,**S**	9.21E-16
19	2,066	2,326	MH765695_PeLCV	MH988458_PaLCV	Unknown (KF307208_PaLCV)	**R**,G,B,M,C,3S	3.22E-12
20	2,326	144	MG757245_ToLCGV	MH765695_PeLCV	KP725055_ToLCV	R,G,M,C,**S**,3S	6.02E-27
21	223	615	HM140366_ChiLCV	MF574143_ChiLC	Unknown (HM140369_PaLCr)	**R**,G,M,C	3.81E-12
22	962	1,539	MH765697_ChiLCV	MH765693_ChiLC	MH765698_ChiLC	**R**,G,M,C,S,3S	4.78E-12
23	1,074	1,555	MH807202_DLCV	MH988458_PaLCV	MH807200_PaLCr	**R**,G,B,M,C,S,3S	5.22E-12
24	438	606	MN529626_PaLCV	Y15934_PaLCV-I	Unknown (HM140367_PaLCr)	**R**,G,B,M,C,S,3S	5.33E-11
25	2,312	2,669	KP725057_AgEV	MH807202_DLCV	JN558352_CLCuM	R,**G**,M,C,3S	9.13E-13
26	1,246	1807	KX302710_PaLCrV	MH674437_PaLCr	Unknown (MH807203_PaLCr)	R,G,M,C,S,**3S**	5.17E-15
27	1,063	1,153	HM140368_PaLCrV	MH765698_ChiLC	Unknown (MH807205_PaLCV)	R,G,M,**3S**	8.02E-11
28	1,554	1785	MH988458_PaLCV	KX302713_PaLCV	MH807202_DLCV	**R**,G,C,S,3S	3.10E-09
29	750	962	MH988458_PaLCV	KX302707_CLCuB	MH765693_ChiLC	**R**,M,C,3S	1.27E-08
30	2,117	2,673	HM140368_PaLCrV	MH807202_DLCV-	Unknown (MH765698_ChiLC)	R,G,C,**S**,S	3.82E-11
31	434	1,072	MN529626_PaLCV	KX302713_PaLCV	KY800906_PaLCV	**R**,G,B,M,S,3S	5.53E-07
32	1,892	2031	KX302713_PaLCV	MN529626_PaLCV	MH807200_PaLCr	**R**,G,M,C,3S	4.97E-08
33	1,108	1,552	HM140365_ChiLCV	Unknown (MH988458_PaLCV)	KX353622_PaYLC	R,G,M,C,**S**,3S	1.27E-15
34	275	440	GU136803_ChiLCV	HM140364_ChiLC	KX353622_PaYLC	R,G,**S**	9.42E-11
35	2,687	26	MH765695_PeLCV	MH765693_ChiLC	Unknown (JN558352_CLCuM)	R,G,S,**3S**	7.35E-07
36	1,279	1,553	MH988458_PaLCV	KX353622_PaYLC	MG757245_ToLCG	R,G,C,**3S**	1.78E-08
37	2,127	2,299	MH807200_PaLCrV	MH988457_PaLCV	KX302707_CLCuB	R,**G**,3S	1.35E-04
38	137	506	Y15934_PaLCV-I	Unknown (MH765698_ChiLC)	MH988458_PaLCV	**R**,G,S,3S	7.21E-05
39	2,651	49	JN558352_CLCuMV	MH765697_ChiLC	KP725057_AgEV	**R**,S,S	1.21E-04
40	1,074	1,223	KP725055_ToLCV	MN529626_PaLCV	Unknown (MH765698_ChiLC)	R,G,M,**C**	6.30E-05
41	302	382	MH988458_PaLCV	MH807205_PaLCV	MH105055_ToLCV	R,G,M,**S**	2.03E-10
42	498	626	MH988458_PaLCV	KX302713_PaLCV	MH807205_PaLCV	**R**,G,M,C,3S	7.02E-04
43	1,390	1,553	MH765698_ChiLCV	MH765695_PeLCV	MH807202_DLCV	R,G,C,**3S**	2.85E-06
44	1,553	1,611	HM140370_ChiLCV	KY800906_PaLCV	Y15934_PaLCV-I	**R**,G,B	2.67E-03
45	1,958	2,051	MH988458_PaLCV	MN529626_PaLCV	MH765697_ChiLC	**R**,M,C,3S	3.69E-03
46	435	959	MH765698_ChiLCV	HM140370_ChiLC	HM140368_PaLCr	R,G,**S**,3S	1.05E-11
47	616	2,106	HM140366_ChiLCV	KY800906_PaLCV	Unknown (MF574143_ChiLC)	R,M,S,**3S**	4.61E-06
48	2,526	2,679	MH988458_PaLCV	Unknown (MH765697_ChiLC)	MH765696_CrYVM	R,C,S,**3S**	5.31E-04
**Betasatellite**							
1	1,053	1,250	MN529627_MM1B	KP725056_CB1/T	MH825686_PSBB3	**R**,G,M,C	2.18E-13
2	580	747	HM143909_Naj1	HM143911_Naj2	Unknown (KX302714_Hajip)	**R**,M,C	8.19E-09
3	1,054	1,159	MH825686_PSBB3	Unknown (KX302716_Guntu)	MH825690_PSBB6	**R**,M,C,S	2.38E-08
4	1,059	213	KX302714_Hajip	MH825689_PSBB6	Unknown (MH825684_PSBB2)	R,**C**,M,S	9.11E-11
5	1,252	170	MH825686_PSBB3	MH825683_PSBB1	Unknown (MH825684_PSBB2)	**R**,G,M,C	8.15E-07
6	576	646	MN529627_MM1B	KP725056_CB1/T	Unknown (MH825691_PSBB6)	**R**,M,C	1.86E-06
7	746	832	KP725056_CB1/T	HM143901_Pani1	Unknown (KX302719_Bhopa)	**R**,M,C	4.74E-06
8	1,090	1,110	KX302716_Guntu	HM143901_Pani1	Unknown (MH825685_PSBB3)	**R**,G,M	6.46E-06
9	27	106	HM143908_PSB8	MH825683_PSBB1	Unknown (KX353620_DP2/T)	**R**,G,M,C	3.07E-05
10	1,157	1,243	KX353620_DP2/T	Unknown (MH825689_PSBB6)	MH825683_PSBB1	R,M,C,**S**,3S	4.32E-05
11	995	1,133	MH825684_PSBB2	KX302717__Moha	MH825685_PSBB3	R,G,**M**,C,S,3S	5.28E-07
12	1,081	1,109	KX353620_DP2/T	Unknown (MH825688_PSBB5)	KP725056_CB1/T	**R**,M,C,S,3S	2.09E-04
13	891	143	KX302717__Moha	MH825689_PSBB6	HM143911_Naj2	R,B,M,**C**	1.82E-06
14	973	1,053	MH825686_PSBB3	MH825689_PSBB6	MH825691_PSBB6	R,G,**S**,3S	1.21E-07
15	1,217	1,259	MH825683_PSBB1	HM143909_Naj1	MH825691_PSBB6	R,G,**S**,3S	1.81E-05

R, RDP; G, Geneconv; B, Bootscan; M, MaxChi; C, Chimarea; S, SiScan; 3Seq, Sequence Triplets; and @The lowest *p*-value calculated for the underline and bold method are given in the column.

**Table 4:** Putative recombination events detected using the RDP4.1 program among genes of *begomoviruses* associated with PaLCD, based on provided datasets from India; six genes/ORF’s of 44 DNA-A sequences.

Event no.	Breakpoints		Recombinant	Parents		Methods	*p* valve
	Begin	End		Major	Minor		
*AV2 (Pre-CP)*							
1	363	85	MN529626_PaLCV	Y15934_PaLCV-I	Unknown (KX302713_PaLCV)	R,G,B,M,C,**S**,3S	9.95E-13
*AV1(CP)*							
1	442	767	MH988458_PaLCV	KX302707_CLCuBV	MH765693_ChiLC	R,G,M,C,S,3S	7.37E-13
2	1	138	KX302713_PaLCV	KX302707_CLCuBV	Unknown (KF307208_PaLCV)	R,G,B,M,C,3S	5.62E-12
3	136	294	MN529626_PaLCV	KY800906_PaLCV	Unknown (JN558352_CLCuM)	R,B,M,C,3S	1.04E-06
4	178	310	HM140366_ChiLC	GU136803_ChiLC	MH674437_PaLCr	R,G,M,3S	6.67E-06
5	244	320	MH988458_PaLCV	MH988457_PaLCV	MH765696_CrYVM	R,G,M,3S	7.82E-05
6	446	165	MH765693_ChiLC	HM140368_PaLCr	MH765695_PeLCV	R,M,C,3S	1.91E-09
*AC3 (Ren)*							
1	87	177	HM140367_PaLCr	MH807203_PaLCr	KF307208_PaLCV	R,G,M,C,S,**3S**	1.88E-11
2	55	151	KX302707_CLCuB	KF307208_PaLCV	JN558352_CLCuM	R,G,M,C,**3S**	8.48E-09
3	403	92	DQ989325_ToLCNDV	MH765697_ChiLC	MH105055_ToLCV	**R**,G,M,C,3S	2.11E-04
*AC2(TrAP)*							
1	235	309	HM140367_PaLCr	HM140369_PaLCr	KF307208_PaLCV	R,G,M,C,S,**3S**	2.18E-15
2	408	217	MH765698_ChiLC	DQ989325_ToLCN	Unknown(MH105055_ToLCV)	R,G,M,C,S,**3S**	1.51E-14
3	215	405	DQ989325_ToLCNDV	MH105055_ToLCV	KY800906_PaLCV	R,G,M,C,S,**3S**	5.83E-15
4	205	405	MG757245_ToLCG	HM140369_PaLCr	KY800906_PaLCV	R,M,C,**S**,3S	1.95E-10
5	52	97	MH807200_PaLCr	MG757245_ToLCG	MH765698_ChiLC	R,M,**3S**	1.52E-03
*AC1(Rep)*							
1	1,072	278	MH988458_PaLCV2	MH807200_PaLCr	Unknown (KP725057_AgEV-)	R,G,M,C,S,**3S**	7.45E-25
2	1,059	283	MH765693_ChiLC2	MH807200_PaLCr	Unknown (KP725057_AgEV)	**R**,G,M,C,S,3S	1.57E-21
3	1,065	276	MH807202_DLCV 1	MH807200_PaLCr	Unknown (KP725057_AgEV)	R,**G**,M,C,S,3S	3.16E-13
4	284	493	MH765693_ChiLC	MG757245_ToLCG	MN529626_PaLCV	**R**,G,M,C,S,3S	2.15E-12
5	274	579	MH807204_PaYLC 1	Unknown (MH807202_DLCV)	HM140368_PaLCr	R,**G**,M,S,3S	1.84E-10
6	279	455	KP725055_ToLCV1	KX353622_PaYLC	Unknown (JN558352_CLCuM)	R,G,M,C,**3S**	9.28E-15
7	680	783	KX302713_PaLCV1	MN529626_PaLCV	MH807200_PaLCr	R,G,M,C,**S**,3S	1.18E-16
8	34	203	JN558352_CLCuM2	KX302707_CLCuB	HM140368_PaLCr	R,M,C,**3S**	3.58E-10
9	271	494	JN558352_CLCuM	Unknown (MH807202_DLCV)	MH807203_PaLCr	R,M,**3S**	3.34E-08
10	1,072	271	KP725057_AgEV2	Unknown(MH765695_PeLCV)	MH807200_PaLCr	**R**,G,M,C,S,3S	1.35E-06
11	281	520	KX353622_PaYLC1	MN529626_PaLCV	Unknown (MH765698_ChiLC)	R,G,M,**3S**	1.69E-10
12	15	278	HM140371_ChiLC1	KX353622_PaYLC	Unknown(DQ989325_ToLCNDV)	R,**M**,C,3S	1.20E-10
13	1,065	286	MH807201_PaLCr1	HM140367_PaLCr	Unknown (MH807200_PaLCr)	**R**,G,M,C,3S	2.81E-05
14	730	1,082	MG757245_ToLCG 1	KP725055_ToLCV	MH765695_PeLCV	R,G,**M**,C,3S	2.22E-07
15	397	473	DQ989325_ToLCNDV 1	Unknown (MH807202_DLCV)	HM140367_PaLCr	**R**,M,C	1.67E-03
16	279	625	MH807203_PaLCr 1	MH807202_DLCV	MH765695_PeLCV	R,M,C,**3S**	1.35E-04
17	751	971	MH988458_PaLCV	MH988457_PaLCV	MH765695_PeLCV	R,G,M,C,**3S**	4.99E-06
18	371	601	MH765695_PeLCV1	MH988457_PaLCV	Unknown(MN529626_PaLCV)	R,**M**,3S	4.63E-04
19	578	785	KP725057_AgEV	HM140365_ChiLC	Unknown (MH765698_ChiLC)	R,M,C,**S**,3S	3.67E-03
*AC4*							
1	127	226	KF307208_PaLCV	Unknown (KP725055_ToLCV)	MH765695_PeLCV	R,M,C,**S**,3S	1.13E-05

R, RDP; G, Geneconv; B, Bootscan; M, MaxChi; C, Chimarea; S, SiScan; 3Seq, Sequence Triplets; and @The lowest *p* value calculated for the underline and bold method are given in the column.

### Coalescent Analysis

An evolutionary scenario signifies the role of nucleotide substitution along with recombination in gaining genetic variation and evolution among *begomoviruses* (Mishra et al., 2020). To detect the nucleotide substitution rate among reference begomoviruses and betasatellites, best-fit substitution models were selected based on the lowest BIC value along with both relaxed and strict molecular clocks. The mean substitution rate among the sequence datasets of DNA-A is 1.83 × 10^−3^ subsite^−1^ year^−1^ [DNA-A, 95% highest posterior density (HPD) interval ranging from 4.126 × 10^−5^ to 6.647 × 10^−3^], which was found to be higher when compared with the range of nucleotide substitution rates of RNA viruses reported so far (Jenkins et al., 2002; Duffy and Holmes, 2009; Kumar et al., 2015), but importantly, the high substitution frequency detected here shows a short-term mutational phenomenon acting on the population rather than a long-term substitution rate. To justify the above, the nucleotide substitution rate was also identified superficially in four gene datasets, i.e., AV2, AV1, AC3, AC2 (Table 5). Additionally, for betasatellites, the mean substitution rate was found to be 1.62 × 10^−6^ subsite^−1^ year^−1^ (*β*, 95% HPD interval ranging from 1.329 × 10^−8^ to 5.08 × 10^−6^). However, a relaxed molecular clock is used to get the suitable value of the mean substitution rate, and the obtained high substitution value is most likely to have resulted from strong positive selection (Duffy and Holmes, 2009). Since then, the population’s selection pressure has been significantly slowed by mutation, which causes codon degeneracy and genetic variation. Therefore, we measured the rate of mutation acting on three nucleotide codon positions, i.e., CP1, CP2, and CP3 respectively, and we found the highest rate of mutation at codon position C3 for DNA-A and among ORFs. The highest mutation rate was found in the AV1 (CP) gene at codon position C3, followed by the other five genes again at the C3 codon position or wobble codon position. Similarly, for betasatellites, the highest mutation rate was found at codon position C1 (Table 5).

**Table 5:** Estimation of Mean Substitution and Codon Degeneracy Rates for DNA-A, ORFs, and betasatellite associated with PaLCD in India.

Viral components	Mean substitution rate (at 95% HPD interval)		Mutation at various codon positions					
	Relaxed clock	Strict clock	Relaxed clock			Strict clock		
			C1	C2	C3	C1	C2	C3
DNA-A	1.8311E-3[4.126E-5, 6.6475E-3]	1.042E-3[6.3439E-4, 1.4373E-3]	0.92	0.91	1.71	0.92	0.90	1.71
Betasatellite	1.6208E-6 [1.3299E-8, 5.0854E-6]	2.2078E-8[1.0648E-26, 1.4473E-7]	1.14	0.94	0.91	1.44	0.94	0.91
AV2(Pre-CP)	1.3048E-3 [3.6509E-4, 2.4095E-3]	1.9376E-3 [1.2877E-3, 2.6304E-3]	1.21	0.54	1.25	1.19	0.55	1.27
AV1(CP)	1.5003E-3 [2.2311E-4, 2.9684E-3]	1.4297E-3 [6.5925E-4, 2.2164E-3]	0.59	0.37	2.04	0.60	0.37	2.04
AC3(Ren)	1.0023E-3[4.1172E-5, 2.2939E-3]	1.5895E-5[1.0495E-27, 1.0621E-4]	1.03	0.97	1.01	1.03	0.97	1.01
AC2(TrAP)	1.2679E-3 [1.5685E-5, 3.4785E-3]	2.7602E-5 [4.9889E-18, 1.7493E-4]	0.66	1.54	1.81	0.66	1.14	1.20
AC1(Rep)	5.8657E-4 [3.3652E-6, 1.5091E-3]	1.1258E-4 [9.3097E-15, 5.2477E-4]	0.84	0.83	1.33	0.85	0.83	1.32
AC4	4.6463E-4[8.113E-15, 1.1734E-3]	2.2745E-3 [3.0007E-4, 4.4365E-3]	0.76	1.77	1.07	0.75	1.76	1.07

### Population Structure

Demography structure analysis was used to estimate the degree of genetic variability (>0.08) within and between populations (Table 6). However, we found the total number of polymorphic sites (s) to be 1757, with a total of 2,808 mutations (η) having a nucleotide diversity of 0.19525 (*π* = 0.1) for DNA-A datasets. Similarly, the analysis was performed for ORF datasets and we found the maximum number of polymorphic sites (s) and number of mutations (η) in AC1 (Rep) and AV1 (CP) genes having nucleotide diversity *π* = 0.1. Moreover, for betasatellite, we found the total number of polymorphic sites (s) 834, with a total 1,482 number of mutations (η) having a nucleotide diversity (*π*) of 0.30509 (*π* = 0.3) was again high. However, the resultant maximum π value explains the non-random distribution of nucleotides throughout viral and sub-viral genome regions, which significantly contributes to a high degree of genetic variability. Therefore, the estimation suggests diverse populations within and among populations.

**Table 6:** Estimation of the Genetic diversity of *begomoviruses* (DNA-A), ORFs, and betasatellites associated with PaLCD in India.

Virus components	*S*	η	*π*	*k*	θ–η	θ–W	*H*	Hd
DNA-A	1,757	2,808	0.19525	510.20085	0.24704	0.15458	43	0.999
Betasatellite	834	1,482	0.30509	330.41176	0.40477	246.69236	17	1.000
AV2(Pre-CP)	213	305	0.13907	47.14315	0.22340	0.15602	31	0.998
AV1(CP)	495	763	0.15251	117.43235	0.22780	0.14778	40	0.989
AC3(Ren)	132	206	0.22713	41.33721	0.26020	0.16673	35	0.968
AC2(TrAP)	123	183	0.17808	37.21882	0.20129	0.13529	37	0.977
AC1(Rep)	579	930	0.12161	181.56342	0.24918	0.15513	42	0.997
AC4	120	206	0.28270	38.16385	0.35079	0.20434	36	0.969

*S, number of segregating sites*; η, *number of mutations*; π, *nucleotide diversity, k, average number of nucleotide differences between sequences.*

*(*θ – η*), Watterson’s estimate of the population mutation rate based on the total number of mutations; (*θ – *w), Watterson’s estimate of the population mutation rate based on the total number of segregating sites; h, number of haplotypes; and Hd, haplotype diversity.*

Further, genetic diversity within and among populations was also determined by the number of haplotypes (H) and haplotype diversity (Hd). Therefore, using DnaSP software (v. 6.0; Rozas et al., 2017; Universitat de Barcelona; origin 1994), analysis was performed for sequence datasets that contained haplotype distribution among reference DNA sequences of the begomovirus population, and we found the total number of haplotypes (H) was 45, and its haplotype diversity was identified as close to 1, i.e., (Hd = 0.99). Simultaneously, among ORF datasets, the number of haplotypes (H) and haplotype diversity (Hd) were found to be distributed between a range of 31–42 and 0.96–0.99, respectively. Thus, the resultant value supports the relative contribution of genes in DNA polymorphism. Similarly, for betasatellites, we found that though the total number of haplotypes (H) is less, i.e., 17, its gene diversity is detected as high, i.e., equal to 1 (1.000). Therefore, the overall result explains the low level of sequence divergence but the high frequency of unique mutations (Table 6).

Neutrality tests were used to assess and understand the demographic selection acting on the genetic population of *begomoviruses* and associated satellite molecules. For evaluation, Tajima’s D test was used, which statistically reflects the negative Tajima’s *D* value for DNA-A datasets at −0.77977, for ORFs range between −0.42227 and −1.45344, and for associated betasatellites at −1.06710. Predominantly, the statistically significant values were negative, which indicates that a large proportion of genetic segregation might be present within sequence datasets that are unique to individual sequences. Similarly, the other parameters, such as Fu & Li’s *D* and Fu & Li’s *F* tests of population statistics, were also evaluated, resulting in negative values for DNA-A, its ORFs, and betasatellites datasets, indicating reiterating of purifying selection and population expansion, which might be due to the inherent diversity. Nevertheless, the combination of Tajima’s D, Fu & Li’s *D*, and Fu & Li’s *F* negative values for DNA-A, its ORFs, and associated satellite population revealed the conserved nature of the gene. Such evidence of nucleotide diversity might be expected when a selective sweep succeeds in the expansion of the population and when most observable segregation functioning at the nucleotide level in a population is momentary and is eventually withdrawn by purifying selection (Table 7).

**Table 7:** Estimation of different neutrality tests for the datasets obtained from identified *begomoviruses* and betasatellites associated with PaLCD in India.

Virus components	Neutrality tests		
	Tajima’s D	Fu & Li’s D	Fu & Li’s F
DNA-A	−0.77977	−0.92210	−1.03898
Betasatellite	−1.06710	--0.65138	−0.89645
AV2(Pre-CP)	−1.45344	−1.91756	−2.08644
AV1(CP)	−1.22580	−1.22580	−1.62944
AC3(Ren)	−0.46638	−0.59617	−0.65493
AC2(TrAP)	−0.42227	−0.39244	−0.48164
AC1(Rep)	−0.55953	−0.61340	−0.70923
AC4	−0.71227	−0.32831	−0.06478

D is the Tajima test statistic estimates neutral mutation hypothesis using DNA polymorphism; Fu and Li’s D* and Fu and Li’s F*: Detecting neutrality of mutations among DNA population.

## Discussion

India shares a large portion of the population that depends on small-area agricultural farming for their subsistence and income. A wide variety of diseases and their infection rates have been seen to cause devastating effects both on crop yield and human persistence. Undoubtedly, the cultivation practises and the presence of tropical climate conditions in the Indian subcontinent boost the prevalence of a large number of plant viruses. *Papaya leaf curl virus* (PaLCuV) is found to infect papaya plants and cause PaLCD, which affects plant growth, fruit size, quality, and quantity, slowing its yield (Shahid et al., 2013; Varun et al., 2017; Bananej et al., 2021), thus accelerating the spread of viral diseases. Additionally, climate change, adaptability, and the fast distribution of vectors and viruses are of major concern for the agriculture sector as they are greatly contributing to the Indian economy.

To explore the evolution of begomoviruses across India, a full-length sequence of reported begomoviruses infecting *Carica papaya* was collected from NCBI and arranged into eight specific datasets containing the sequence of DNA-A, its six ORFs, and associated betasatellites. Using the MEGA X program, we calculated the best-fit nucleotide substitution model for further analysis. i.e., (GTR + G) for DNA-A, ORFs (AV1:TN93 + G + I); (AV2: HKY + G); (AC1& AC3: GTR + G); (AC2:TN93 + G); (AC4: HKY + G); and (TN93 + G + I) for betasatellites. The recurrent occurrence of recombination and nucleotide substitution alike in RNA viruses is mostly attributed to factors contributing to the high degree of genetic variability among *begomovirus* populations, which may significantly step up their evolution by expanding the combinations of pre-existing nucleotide segregation created by mutation (Duffy and Holmes, 2008, 2009). Accordingly, recombination and mutation are often stated as the chief contributors to genetic variability, which is the subject matter of investigation in the present study using molecular and computational efficacy.

The phylogenetic results revealed homological diversification and the mean branch length observed for DNA-A and betasatellites datasets shows the possibility of a recombinational event in the studied population of begomovirus (Lima et al., 2017). Thus, the phylogenetic dendrogram signifies the evolution of the begomoviruses with the papaya host, which strongly encourages the coadaptation of associated betasatellites. We further compared the result of the maximum likelihood phylogenetic tree from MEGA X software with the maximum clade credibility phylogenetic tree from BEAST. We found that the ML tree shows very similar topologies to the maximum clade credibility phylogenetic tree, with many nodes in the DNA-A tree receives both strong bootstrap support and high posterior probabilities, and with respect to clade formation, they are found to be similar as both trees form four major groups (Supplementary Figure S1). To exclude other biases that strengthen the significant differences between the degrees of intra and inter-species variability, we checked for different rates of transition and transversion substitutions and transition/transversion bis (R). Pervious literature states that C → T and G → A are the most common base substitution transitions in the AC4 and AV2 regions, whereas A → T, A → C, and G → T transversions are more common in the IR than in the other four regions (Begun et al., 2007; Duchêne et al., 2015). However, our results indicate that, under the conditions studied, begomovirus AV1 and AC2 genome regions are more prone to transition substitution mutations and accept more drastic amino acid changes than other genomic regions. Therefore, the estimation suggests that the role of base substitutions causes transition mutation at a higher rate in a population than single nucleotide polymorphism (Sánchez et al., 2018; Mishra et al., 2020).

The phylogeny-based partitioning method was qualitatively estimated for eight datasets, resulting in mean branch length, and was found useful in quantifying the effect of recombination events. Previous studies have revealed that recombination happens at high frequencies in *begomovirus* populations that use a conserved feature, i.e., a rolling circle mechanism for replicating their genomes and making them mechanistically recombination-prone, thus generating recombination breakpoints in a non-random location (Martin et al., 2011). As recombination rates are threatened for plant viruses, our experimental analysis detected a recombination event in the genomic region of eight datasets using different algorithms of RDP 4.1 software (Martin et al., 2015), showing variable major and minor parents causing an uneven distribution of recombination breakpoints, leading to genetic diversity. The AC1 gene region of the genome sequence shows the maximum number of recombination break points within nucleotide positions 1,527–2,612, followed by the AV1 gene, and the least recombination break points were observed in the AC4 region of the gene. The distribution of events was maximally detected among intra-species isolates and the least was found between inter-species isolates. Additionally, for betasatellites, a low recombination breakpoint is detected in βC1 region within nucleotide position 200–562. This indicates that the tolerant capacity could be greater for some portions of the genome towards recombination events than others, involving more divergent parental viruses (Martin and Williamson, 2005). However, the statistically measurable recombination rate seems to be lower than the mutation rate in sequence datasets. Even in such cases, recombination events act actively, but consequently, the mutation dynamics were found to be a leading force in shaping the standing genetic variability (Kumar et al., 2015).

Significant aspects of population genetics are possibly accompanied by mutation, along with recombination, neutral selection, genetic drift, and gene flow, which encourage shaping the genetic structure of populations (Xavier et al., 2021). We calculated the rate of nucleotide substitution using coalescent best fit substitution modal with strict and relaxed molecular clocks for each dataset and found the relaxed molecular clock to be promising for having a high mean substitution rate for DNA-A, ORFs, and betasatellite. However, this condition could possibly be influenced by either spontaneous mutations or the usage of error-prone DNA polymerases in virus replication (Richter et al., 2016). To find out whether our estimates of substitution rate are robust and contain clear temporal structure, we randomly reshuffled the dates of isolation for viruses in each of the eight datasets and reran BEAST using the best-fitting parameters from the actual analyses. For five datasets, i.e., AV1, AC1, AC2, and AC3 genes and betasatellites, the reshuffled control 95% HPD values excluded the mean nucleotide substitution rates estimated from the actual data, and for the remaining three datasets, i.e., DNA-A, AV2, and C4 genes, the case is different in that the reshuffled results were superimposed on the substitution rates from the real data, indicating that there was insufficient temporal structure to reliably estimate substitution rates. Importantly, all these datasets were sampled over a 21-year span. In contrast, the AV1, AC1, AC2, and AC3 genes and betasatellites had more distinct substitution rate estimates from their reshuffled controls, reflecting a clear temporal signal (Supplementary Figure S2). Moreover, we also identified codon position C3 to be significantly affected by the selection pressure acting on the population caused by mutation.

Additionally, it is important to address the key issue that refers to the uneven distribution of variation across *begomovirus* genomes. In this context, the combination of various factors is responsible for affecting genetic variability and acts by distributing polymorphisms in a non-random manner in the genomic regions of *begomoviruses* (Mishra et al., 2020). However, we calculated the degree of genetic variability and haplotype diversity within and among populations by using several parameters of DnaSP software (v. 6.0) and found a high degree of genetic variability. The resulting data explain the non-random distribution of nucleotides throughout viral and sub-viral genome regions, resulting in population diversity (Sobrinho et al., 2014). Further, haplotype diversity indicates the low level of sequence divergence but high frequency of unique mutations, causing uniqueness within and among populations.

Moreover, to understand the population genetics and selection forces acting on the begomovirus population and satellite molecules, neutrality tests were evaluated, resulted in a negative value, suggesting that populations might be influenced by purifying selection or have experienced recent expansion (Mishra et al., 2020) rather than neutral selection. Nevertheless, the combination of Tajima’s D, Fu & Li’s *D*, and Fu & Li’s *F* negative values for DNA-A, its ORFs, and associated satellite population revealed the conserved nature of the gene. Such evidence of nucleotide diversity might be expected when most observable segregation functioning at the nucleotide level in a population is momentary and is ultimately withdrawn by purifying selection. Our results indicate that *begomoviruses* infecting *Carica papaya* are not restricted to any solitary geographical region of India.

Although the number of sequence data for DNA-A, betasatellites, and particularly alphasatellites is insufficient, the current study’s findings may provide meaningful basic information that contributed significantly to the diversification of begomoviruses, particularly those causing PaLCDs, and acknowledge the evolutionary potential, particularly in the context of recombination, adaptation, genetic diversity, emergence, and evolution.

## Conclusion

The present study, based on bioinformatics approaches, provides the current understanding of the genetic diversification of the begomovirus population associated with PaLCD. The coexistence of the begomovirus population and its satellite molecules, as explained by geographical distribution, indicates their interdependence in accelerating disease severity and spread among economically important crops such as papaya across India. A wide begomovirus population having a common host might be attributed to an uncontrolled evolutionary variation in its DNA genome, which is mainly driven by a high frequency of mutational changes. Furthermore, the variable frequency of mutation demonstrated the random allocation of the recombination breakpoints, leading to a diversified distribution of recombinational patterns. Moreover, this phenomenon uses different mechanisms to overwhelm selection pressure and to effectively adapt to environments with new hosts. Thus, that geographically separates begomovirus and its associated satellites. The continuous evolution of new recombinants and their satellites might lead to efficient vector transmission, expansion of host range, and breaking of resistance, which poses a threat to crop production at an alarming rate, thus affecting the agroeconomic value and disease management. However, for expanding knowledge in an area, the depth study of molecular fundamentals together with vector-mediated and host-dependent dispersal might provide an understanding of the expanding begomovirus disease complex.

## Data Availability Statement

The original contributions presented in the study are included in the article/Supplementary Material; further inquiries can be directed to the corresponding authors.

## Author Contributions

AS and VP performed experiments and wrote the manuscript. AKS helped in data collection. RG and DY guided the design of the whole test scheme, and AA-S and MS critically reviewed the manuscript. All authors contributed to the article and approved the submitted version.

## Conflict of Interest

The authors declare that the research was conducted in the absence of any commercial or financial relationships that could be construed as a potential conflict of interest.

## Publisher’s Note

All claims expressed in this article are solely those of the authors and do not necessarily represent those of their affiliated organizations, or those of the publisher, the editors and the reviewers. Any product that may be evaluated in this article, or claim that may be made by its manufacturer, is not guaranteed or endorsed by the publisher.

## Footnotes

^1^ www.ncbi.nlm.nih.gov

## Supplementary Material

Supplementary Figure S1 Maximum clade credibility phylogenetic tree associated with papaya leaf curl disease begomoviruses in India aligned using BEAST software v.1.10’s FigTree program. Tree is formed using the relax.tree file, which is automatically rooted by the use of a relaxed molecular clock.

Supplementary Figure S2 Graphical representation of a temporal signal generated by the BEAST software; Estimated rates of nucleotide substitution from actual date of isolation for begomoviruses, ORFs, and betasatellites associated with PaLCD (eight datasets) with 95% HPD values, are shown in dark grey. The reshuffled mean estimated rates for eight datasets with 95% HPD values are shown in light grey.
