# Supplementary figures and images for: Evolutionary Dynamics of Begomoviruses and Its Satellites Infecting Papaya in India
Source: Front Microbiol. 2022 May 12;13:879413. doi: 10.3389/fmicb.2022.879413 (PMC9171567; doi:10.3389/fmicb.2022.879413)

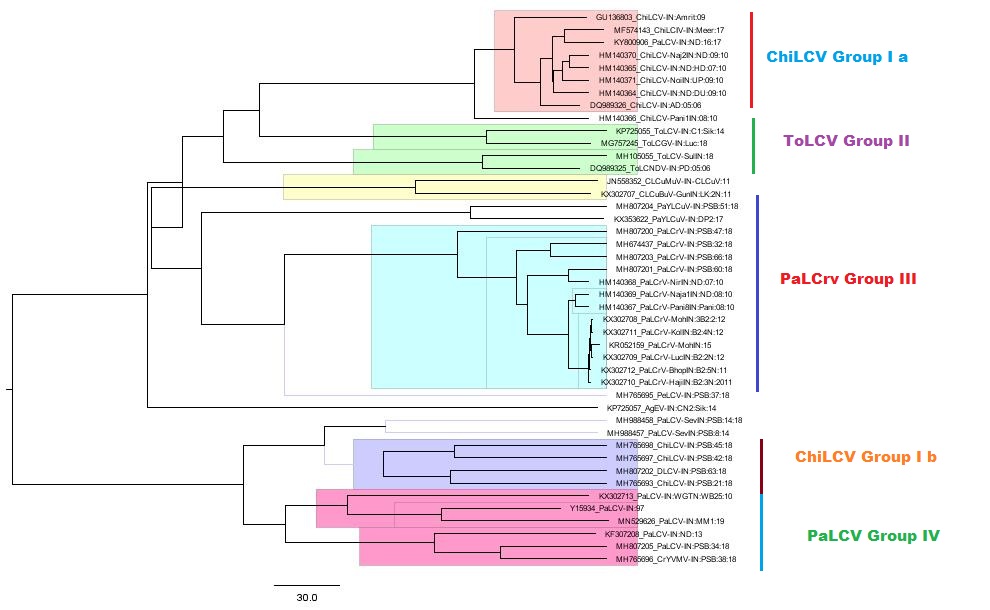

Supplement: Supplementary Figure S1 — Maximum clade credibility phylogenetic tree associated with papaya leaf curl disease begomoviruses in India aligned using BEAST software v.1.10’s FigTree program. Tree is formed using the relax.tree file, which is automatically rooted by the use of a relaxed molecular clock. [file Image_1.JPEG]

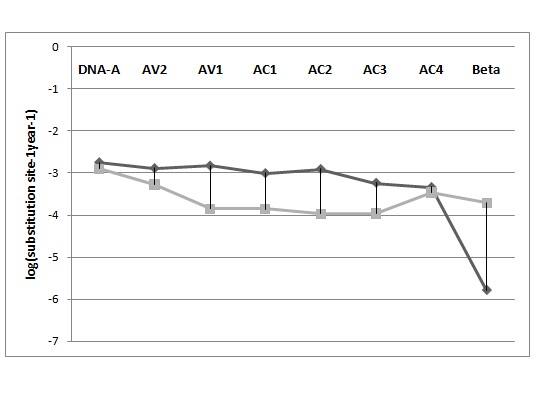

Supplement: Supplementary Figure S2 — Graphical representation of a temporal signal generated by the BEAST software; Estimated rates of nucleotide substitution from actual date of isolation for begomoviruses, ORFs, and betasatellites associated with PaLCD (eight datasets) with 95% HPD values, are shown in dark grey. The reshuffled mean estimated rates for eight datasets with 95% HPD values are shown in light grey. [file Image_2.JPEG]
